# Combined Small RNA and Degradome Sequencing Reveals Novel MiRNAs and Their Targets in the High-Yield Mutant Wheat Strain Yunong 3114

**DOI:** 10.1371/journal.pone.0137773

**Published:** 2015-09-15

**Authors:** Feng Chen, Xiangfen Zhang, Ning Zhang, Shasha Wang, Guihong Yin, Zhongdong Dong, Dangqun Cui

**Affiliations:** 1 Agronomy College/Collaborative Innovation Center of Henan Grain Crops/National Key Laboratory of Wheat and Maize Crop Science, Henan Agricultural University, Zhengzhou, China; 2 Zhoukou Academy of Agricultural Sciences, Zhoukou, China; East Carolina University, UNITED STATES

## Abstract

Wheat is one of the main food sources worldwide; large amount studies have been conducted to improve wheat production. MicroRNAs (miRNAs) with about 20–30 nucleotide are a class of regulatory small RNAs (sRNAs), which could regulate gene expression through sequence-specific base pairing with target mRNAs, playing important roles in plant growth. An ideal plant architecture (IPA) is crucial to enhance yield in bread wheat. In this study, the high-yield wheat strain Yunong 3114 was EMS-mutagenesis from the wild-type strain Yunong 201, exhibiting a preferable plant structure compared with the wild-type strain. We constructed small RNA and degradome libraries from Yunong 201 and Yunong 3114, and performed small RNA sequencing of these libraries in order identify miRNAs and their targets related to IPA in wheat. Totally, we identified 488 known and 837 novel miRNAs from Yunong 3114 and 391 known and 533 novel miRNAs from Yunong 201. The number of miRNAs in the mutant increased. A total of 37 known and 432 putative novel miRNAs were specifically expressed in the mutant strain; furthermore, 23 known and 159 putative novel miRNAs were specifically expressed in the wild-type strain. A total of 150 known and 100 novel miRNAs were differentially expressed between mutant and wild-type strains. Among these differentially expressed novel miRNAs, 4 and 8 predict novel miRNAs were evidenced by degradome sequencing and showed up-regulated and down-regulated expressions in the mutant strain Yunong 3114, respectively. Targeted gene annotation and previous results indicated that this set of miRNAs is related to plant structure. Our results further suggested that miRNAs may be necessary to obtain an optimal wheat structure.

## Introduction

MicroRNAs (miRNAs), a class of regulatory small RNAs (sRNAs), are a large family of endogenous non-coding RNAs with a length of approximately 20 nucleotide (nt) to 24 nt; miRNAs regulate gene expression by sequence-specific base pairing with target mRNAs [1]. miRNAs account for approximately 1% of the predicted genes in higher eukaryotic genomes and may modulate a range from 10% to 30% of genes [2, 3]. miRNAs are derived from a hairpin precursor sequence with approximate 70 bases; this sequence is transcribed into a single-stranded primary microRNA (pri-miRNA) by RNA polymerase II [4]. Pri-miRNAs undergo nuclear cleavage to form precursor microRNAs (pre-miRNAs), which are then processed by dicer-like 1 to form a 21 bp double-stranded RNA duplex with a conserved stem and variable loops; this duplex is subsequently excised to produce mature miRNA [5]. Mature miRNA is incorporated into an RNA-induced silencing complex (RISC) to target mRNAs for cleavage in a sequence-specific manner [6, 7]. A miRNA sequence can have multiple targets, and plant miRNAs recognize their targets by near-perfect complementarity to direct an RISC-mediated cleavage.

Approximately 25,000 miRNAs have been identified in 193 species of animals, plants, and microorganisms [8], playing fundamental roles in development, biological function, and maintenance of tissues and cells [9]. miRNAs mainly down-regulate gene expression in different processes, such as translational repression, mRNA cleavage, and epigenetic modification. In plants, miRNAs are implicated in several biological processes during plant development [10, 11], such as leaf and floral organ development, lateral root formation, hormone signaling, cell differentiation and proliferation, cell death, and signal transduction in response to stress [1, 12, 13]. Investigating the biological functions of miRNAs, identifying their targets, and analyzing their regulatory mechanisms are important to understand life, health, and disease.

Wheat (*Triticum aestivum* L.) is one of the most important crops worldwide, providing approximately 20% of human dietary calories [14]. Therefore, wheat has been genetically improved to enhance quality and yield and to adapt various environments and tolerate biotic stresses for global consumption. Plant structure significantly affects grain yield in bread wheat and directly regulates the number of ears per unit area and panicle length [15]. Therefore, ideal plant architecture (IPA) in crops has been developed. Several reports have evidenced that IPA is necessary to regulate rice plant structure, thereby enhancing grain yield [16–18]. The molecular mechanisms that regulate plant growth stages should be elucidated to improve plant architecture. miRNAs are involved in these improvement processes, which would significantly contribute to human welfare [19].

High-technology approaches, such as next-generation sequencing platforms and bioinformatics prediction, have provided large-scale programs for plant genomic analysis. For example, transcriptome-wide experimental method called “degradome sequencing” or “parallel analysis of RNA ends (PARE)” has been used to directly and globally identify the residues of small RNA-directed target cleavage [20–22]. Another example is high-throughput miRNA profiling combined with degradome sequencing, in which miRNA and mRNA cleavage profiles are integrated; with this combined technology, the depth of miRNA cloning coverage and the pace of miRNA identification have been remarkably expanded in plants [20]. Studies have suggested that whole-scale miRNA analysis can help discover regulatory networks in plants [23]; moreover, specific miRNA identification and target analysis may reveal key signaling components that regulate plant metabolism, growth, and development [24–26]. miRNA profiling by next-generation sequencing technologies can be applied to identify novel and known miRNAs [27–29]; however, only few studies have employed degradome sequencing to identify miRNA-directed mRNA cleavage in rice [30–32], maize [33, 34], and wheat [35–37]. Therefore, studies have mainly focused on gene regulation of miRNA on their targets by degradome sequencing.

Efforts have been devoted to unravel regulatory events that control responses to abiotic stresses; for example, miR156 and miR398 are upregulated in response to drought whereas miR165/166, miR170/171, and miR396 are down-regulated [12]. Furthermore, miRNA profiles present different miRNA induction kinetics between heat-tolerant and heat-susceptible cultivars under heat stress (40°C) [38]. However, limited information is available regarding the miRNA-mediated developmental regulation of plant architecture and high yield. Yunong 3114 strain is a high-yield mutant wheat strain derived from the high-quality noodle wheat strain Yunong 201; Yunong 3114 exhibits a relatively preferable plant architecture for wheat breeding program. The transcriptome responsible for high yield was analyzed in our previous work. In the present study, small RNA populations were subjected to high-throughput degradome sequencing and bioinformatics analysis to identify miRNAs that are potentially involved in the developmental regulation of plant architecture. Using a combined sequencing strategy, we identified a total of 83 new miRNAs by small RNA and degradome sequencing from small RNA library and degradome library of wheat. Degradome analysis results showed that 304 putative target genes were predicted for 96 miRNAs in mutant strain, whereas 300 genes were targeted by 73 miRNAs in wild strains. Our study provided information to investigate the plant architecture associated miRNAs and the interactions with their targets.

## Materials and Methods

### Materials and cultures

**Fig 1:**
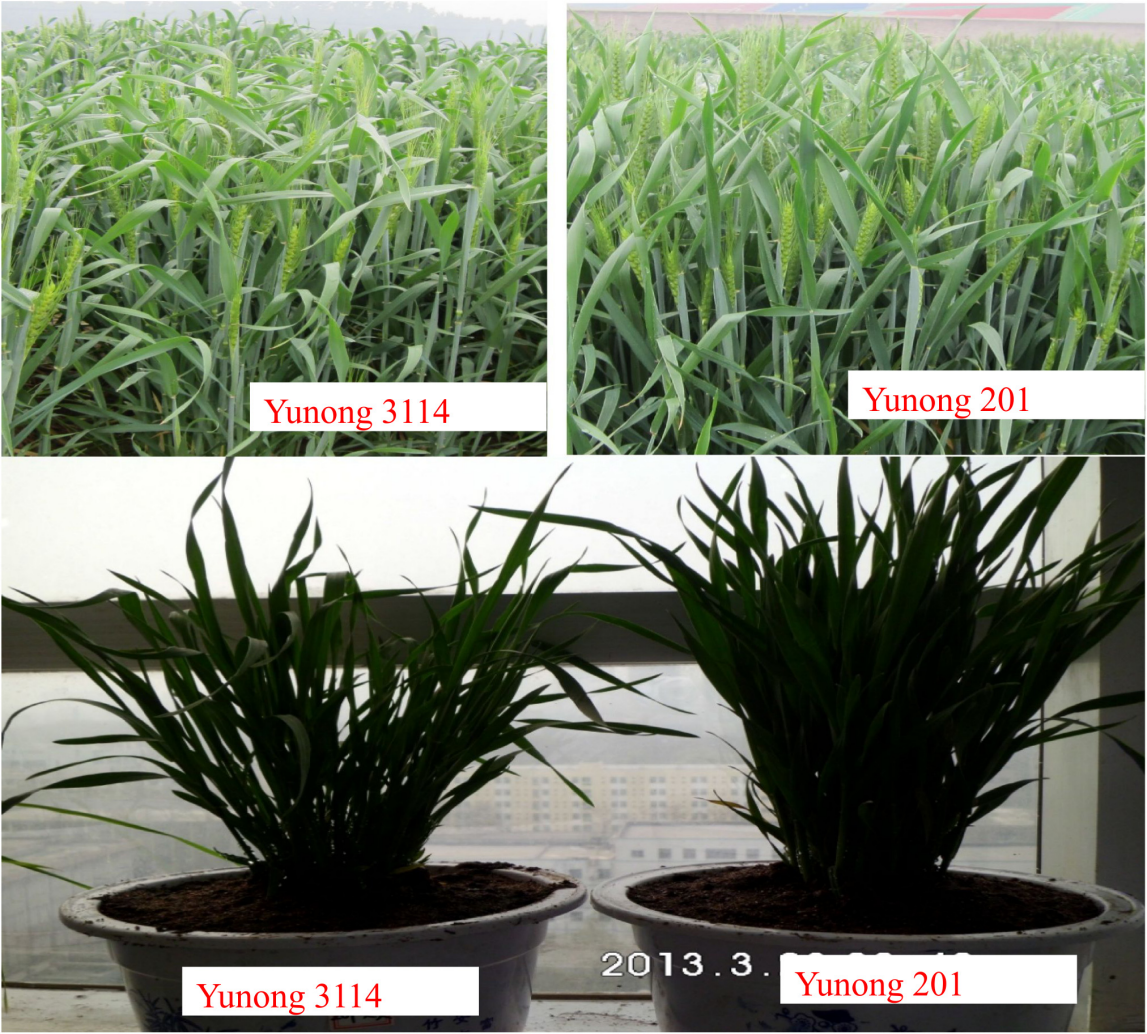
Comparison of plant architectures of Yunong 3114 and Yunong 201 in field and green house.

Chinese winter wheat cultivar Yunong 201, a high-quality noodle wheat cultivar with outstanding dry white noodle quality, was developed in 2006. In the present study, the cultivar was treated with 0.8% ethyl methanesulfonate (EMS). An M_2_ line with different plant architectures and larger kernel sizes was screened from the EMS-mutagenized population of Yunong 201. This line was subsequently self-crossed four times into an M_6_ line Yunong 3114 (Fig 1). Yunong 201 and its derived line Yunong 3114 were planted and grown at the Zhengzhou Scientific Research and Education Center of Henan Agricultural University (N34.9; E113.6) in cropping seasons in October 2010 under non-stressed conditions under local management practices. Each plot was comprised of four 200-cm long rows with 23 cm between neighboring rows and 10 cm between neighboring plants. All surveyed plants were vernalized through winter with an average temperature of 1.5°C (December, January, and February) in 2011. After one week of wheat blooming in April 2011, ears, leaves, stems, and roots in the booting stage of three plants of each strain were rapidly collected and then mixed on the basis of their weights for each plant. These tissues of each plant were used to generate small RNA libraries of Yunong 201 and Yunong 3114; and the same amount of small RNA of three plants of each cultivar were mixed and were generated into two small RNA libraries. These two libraries were then delivered to Oebiotech Company (Oebiotech, Shanghai, China) to determine and analyze miRNA.

Before harvesting, ten plants of each variety were randomly selected in each plot and used to determine tiller angle, number of effective tillers per plant, plant height, flag leaf angle, flag leaf open angle, flag leaf length, flag leaf width, and flag leaf area (calculated from the product of leaf length and maximum leaf width * 0.75). At harvest, grain number per spike, grain weight per spike, and grain weight per plant were determined.

### Construction of small RNA and degradome libraries

Small RNA library construction and deep sequencing were performed according to the method of Malone et al. [39]. In brief, 50% polyethylene glycol was used to separate low-molecular weight RNA from total RNA to obtain a concentration of 5%. Subsequently, 5 M NaCl was used to finalize the concentration to 0.5 M. Small RNAs (18 nt to 30 nt) were purified by PAGE and then ligated to 5′ and 3′ oligonucleotide RNA adaptors. Reverse transcription and polymerase chain reaction (PCR) were also performed. PCR products were purified and sequenced using an Illumina/Solexa sequencing by synthesis technology (Oebiotech, Shanghai, China).

Degradome library was constructed using the pooled total RNA from the ear, leaf, stem, and root [21, 30]. In brief, poly (A) RNA was extracted and ligated to 5′ RNA adapter. The products were digested after RT-PCR and ligated to 3′ dsDNA adapter. The amplified library was subjected to gel purification and then sequenced with an Illumina/Solexa Hiseq 2000 platform (Oebiotech, Shanghai, China).

### Bioinformatics analysis of sequencing data

Small RNA reads and degradome reads were generated by Illumina/Solexa HiSeq™ analysis (Oebiotech, Shanghai, China). The basic reads were converted into sequence data (also called raw data/reads) by base calling. Low-quality reads were filtered, and the reads with 5′ primer contaminants and poly (A) were removed. The reads without 3′ adapter and insert tag, the reads shorter than 18 nt from the raw data, and the clean reads were obtained. For primary analysis, the length distribution of the clean sequences in the reference genome was determined. Non-coding RNAs were annotated as rRNAs, tRNAs, small nuclear RNAs (snRNAs), and small nucleolar RNAs (snoRNAs). These RNAs were aligned and then subjected to the BLAST search against Rfam v.10.1 (http://www.sanger.ac.uk/software/Rfam) and GenBank databases (http://www.ncbi.nlm.nih.gov/genbank/). The known miRNAs were identified by aligning against an miRBase v.18 database (http://www.mirbase.org/), and the known miRNA expression patterns in different samples were analyzed. Unannotated small RNAs were analyzed by MIREAP (https://sourceforge.net/projects/mireap/) to predict novel miRNAs. Conserved and novel miRNA precursor sequences were identified using MIREAP software, which was developed by BGI on the basis of the transcriptome sequences of Yunong 3114 and Yunong 201 (previous data). Based on the hairpin structure of a pre-miRNA and the miRBase database, the corresponding miRNA star sequence was also identified.

For degradome sequencing data, clean reads were generated using the method performed in small RNA sequencing analysis. The filtered reads were then aligned to the reference genome by SOAP [40] to identify the fragments of degraded mRNAs. The alignments against Rfam v.10.0 and GenBank databases were sequentially performed. Subsequently, the miRNA-mRNA pairs for known and novel miRNAs identified from small RNA sequencing were subjected to PairFinder software [41]. Similarities with an E value lower than e^−10^ was considered as positive hits.

### Functional analysis of target genes

Functional annotation of the predicted target genes of miRNAs was performed using a gene ontology system by AmiGO program (amigo.genontology.org) to describe biological process. The pathways and molecular interaction network of the predicted target genes were evaluated using KEGG (www.genome.jp/kegg).

### Real-time quantitative RT-PCR

Total RNA was extracted from wheat Yunong 3114 and Yunong 201 using a miScript II reverse-transcription kit (Qiagen, Germany) according to the manufacturer’s specifications. Real-time PCR was performed using a LightCycler® 480 II real-time PCR instrument (Roche, Swiss). The reactions were incubated in a 384-well optical plate (Roche, Swiss) at 95°C for 10 min and then subjected to 40 cycles of 95°C for 10 s and 60°C for 30 s. Each sample was run in technical triplicate for analysis, and each experiment was biological repeated three times. miRNA-specific primer sequences were designed in our laboratory and synthesized by Generay Biotech (Generay, PRC) on the basis of the miRNA sequences obtained from the miRBase v.20.0 database. The miRNA-specific primer sequences are presented in S1 Table. The expression levels of miRNAs were normalized to the reference gene 5S rRNA and then calculated using the 2^-ΔΔCt^ method [42].

## Results

### Overview of small RNA library sequencing

The high-yield mutant wheat Yunong 3114 was obtained from the EMS-treated Yunong 201. The plant architecture of Yunong 3114 was significantly different from that of Yunong 201 in terms of size and type of flag leaves, number of ears per unit area, and tiller angles (Fig 1 and Table 1). Tiller angle is a key trait contributing to ideal plant architecture and higher grain yield [18]. In our result, the tiller angle of the mutant strain (25.3°) was larger than that of wild strain (15.2°), and also the flag leaf open angle (80.6° vs. 45.5°) and flag leaf area (46.2 vs. 40.1 cm^2^) were both larger in the mutant strain. The thousand kernel weight of mutant strain was heavier than the wild strain (46.8 vs 42.3 g) (Table 1). We focused on IPA because of its positive effect on high yield.

**Table 1:** Comparison of agronomic traits of Yunong 201 and Yunong 3114.

Traits	Yunong 201	Yunong 3114
**Tiller angle**	15.2°	25.3°
**Effective Tillers Per Plant**	14.3°	17.5°
**Flag leaf angle**	40.2°	40.3°
**Flag leaf open angle**	45.5°	80.6°
**Flag leaf length (cm)**	24.5	26.8
**Flag leaf width (cm)**	2.18	2.30
**Flag leaf area (cm** ^**2**^ **)**	40.1	46.2
**Plant height (cm)**	84.2	81.8
**Kernel length (cm)**	6.8	7.5
**Kernel width (cm)**	3.14	3.12
**Thousand kernel weight (g)**	42.3	46.8

**Fig 2:**
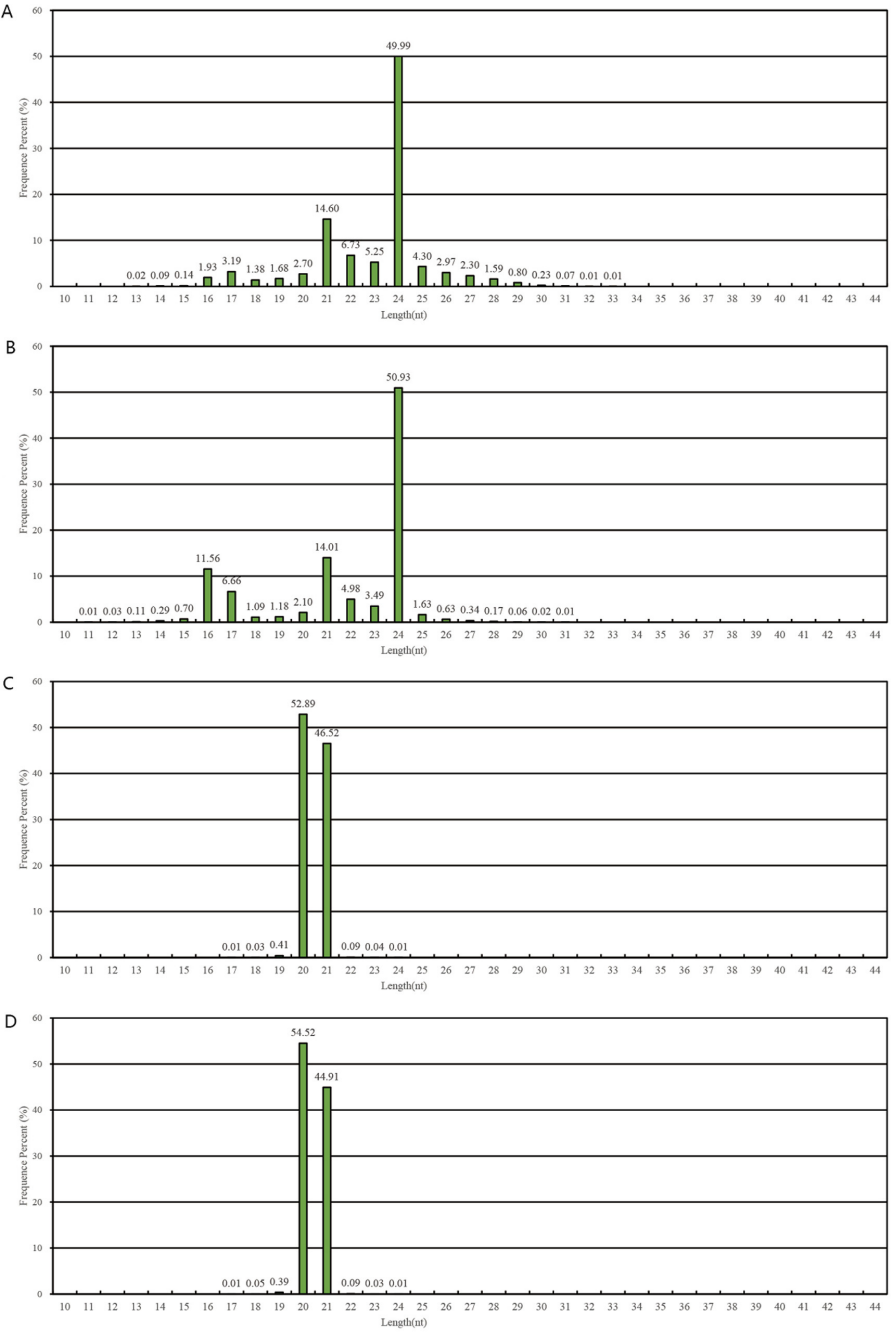
Distribution of fragment lengths in wild and mutant wheat strain by small RNA sequencing and degradome sequencing. A) wild strain by small RNA sequencing; B) mutant by strain small RNA sequencing; C) wild strain by degradome sequencing; D mutant strain by degradome sequencing.

To study the role of miRNAs in regulating the plant architecture of wheat, we isolated the total RNA of wild-type and mutant wheat strains. Afterwards, they were subjected to Illumina/Solexa high-throughput RNA sequencing to obtain miRNA expression profiles. A total of 10,822,238 total reads were obtained from wild strain Yunong 201 between 13 and 33 nt in length. Low-quality reads, 4549 reads for 3′ adapter, and 95,431 reads for 5′ contaminant were eliminated; thus, 10,107,814 clean reads were obtained in the wild-type sample (Table 2). Two peaks at 21 and 24 nt were observed in the length distribution data (Fig 2A). Moreover, 13,859,834 total reads were obtained from mutant strain Yunong 3114 between 13 and 33 nt in length. After eliminating the low-quality reads, 11,024,149 clean reads were obtained in the mutant sample (Table 2). In contrast to the peaks of the wild-type sample, four peaks at 16, 17, 21, and 24 nt were observed in the length distribution data (Fig 2B). These differences in the complexity of the two small RNA pools indicated differential regulation of gene expression in the two wheat strains.

**Table 2:** Data set summary of sequencing of two (wild and mutant strain) small RNA and degradome libraries.

	Category	Wild		Mutant	
		Number	Percentage (%)	Number	Percentage (%)
**Small RNA data**	Total reads	10822238		13859834	
	High quality	10789735	100%	13823204	100%
	3'adapter null	4549	0.04%	4057	0.03%
	Insert null	7170	0.07%	9537	0.07%
	5'adapter contaminants	95431	0.88%	138265	1.00%
	Smaller than18nt	574575	5.33%	2646760	19.15%
	polyA	196	0.00%	436	0.00%
	Clean reads	10107814	93.68%	11024149	79.75%
**Degradome data**	Total reads	19013429		19013429	
	High quality	18962247	100%	18962247	100%
	3'adaptor null	8405	0.04%	8405	0.04%
	Insertnull	17	0.00%	17	0.00%
	5'adaptorcontaminants	70405	0.37%	70405	0.37%
	Smaller than 18nt	3160	0.02%	3160	0.02%
	Clean reads	18880260	99.57%	18880260	99.57%

Approximately 69.76% and 64.74% of the total sRNA corresponded to 60.05% and 57.21% of unique sRNA in the mutant and wild-type libraries, respectively; the total sRNAs were mapped onto the reference genome (Table 3) using SOAP software. All these sRNAs were analyzed against Genebank and Rfam databases to remove the known rRNA, tRNA, and small nuclear RNA (snRNA) (S2 Table). Moreover, seven categories, namely, miRNA, rRNA, snoRNA, snRNA, repeat-associated sRNA, tRNA, and degraded fragments of mRNA introns or exons, were annotated (Table 4). The structural sRNAs accounted for approximately 18.8% of the total sRNA (17.31% of the unique sRNA) in mutant strain, whereas 22.8% of the total sRNA (17.2% of the unique sRNA) in wild strain. The remaining sequences were mapped to miRBase and MIREAP to identify and predict known and novel miRNAs. We speculated that small RNAs were low in abundance but highly diverse. The mutant wheat contained higher fractions of total and unique sRNAs than the wild-type wheat; this result suggested that EMS-induced mutation caused these differences in the two strains.

**Table 3:** Distribution of reads in the reference genome.

	Categories	Unique sRNAs (%)		Total sRNAs (%)	
		wild	mutant	wild	mutant
**Small RNA data**	Total sRNAs	3339160 (100%)	4215523 (100%)	10107814 (100%)	11024149 (100%)
	Matched sRNAs	1910184 (57.21%)	2531279 (60.05%)	6543925 (64.74%)	7690084 (69.76%)
**Degradome data**	Total sRNAs	7876103 (100%)	4226883 (100%)	20890799 (100%)	18880260 (100%)
	Matched sRNAs	5246562 (66.61%)	2881890 (68.18%)	15983212 (76.51%)	13865266 (73.44%)

**Table 4:** Classification of annotation sRNAs.

Categories	Unique sRNAs (%)		Total sRNAs (%)	
	Wild	Mutant	Wild	Mutant
**Total**	3339160 (100%)	4215523 (100%)	10107814(100%)	11024149 (100%)
**miRNA**	24923 (0.75%)	24335 (0.58%)	945274 (9.35%)	1104279 (10.02%)
**rRNA**	96983 (2.90%)	55415 (1.31%)	981075 (9.71%)	480922 (4.36%)
**repeat**	458769 (13.74%)	663790 (15.75%)	1099884 (10.88%)	1456763 (13.21%)
**snRNA**	2770 (0.08%)	1791 (0.04%)	10919 (0.11%)	5375 (0.05%)
**snoRNA**	774 (0.02%)	500 (0.01%)	2355 (0.02%)	1255 (0.01%)
**tRNA**	15303 (0.46%)	8402 (0.20%)	210282 (2.08%)	128992 (1.17%)
**unannotated**	2739638 (82.05%)	3461290 (82.11%)	6858025 (67.85%)	7846563 (71.18%)

### Identification of known miRNAs in the mutant strain Yunong 3114 and wild-type strain Yunong 201

To identify known miRNAs in the two wheat strains, we aligned the small RNA sequences with the known miRNA precursors and mature miRNAs from plants in the miRBase. Overall, the known wheat miRNAs accounted for 10.02% of the total sRNAs and 0.58% of the unique sRNAs in the mutant library; by contrast, 9.35% of the total sRNAs and 0.75% of the unique sRNAs were found in the wild-type library (Table 4). The mutant wheat had lower unique sRNAs but higher total sRNAs than the wild-type wheat, indicating that mutation generated more known miRNAs but lower unique sRNAs. Moreover, 488 known miRNAs in the mutant library and 391 in the wild-type library were detected by sRNA sequencing. For example, miR166, miR167, miR168, miR159, miR528, and miR5062 exhibited a relatively high expression level across the two small RNA libraries (>25,000 each, data not shown). To predict precursor, we used the transcriptome sequences of Yunong 3114 and 201 (previous unpublished data) and obtained inverted repeats and stem-loop structures. Only the sequences that perfectly matched with known plant miRNA mature sequences or with miRNA precursors were considered as conserved miRNAs. All of the 147 conserved families were included, indicating that small RNA sequencing data showed a good coverage of the known miRNAs.

### Prediction of novel miRNAs in wheat Yunong 3114 and 201

**Fig 3:**
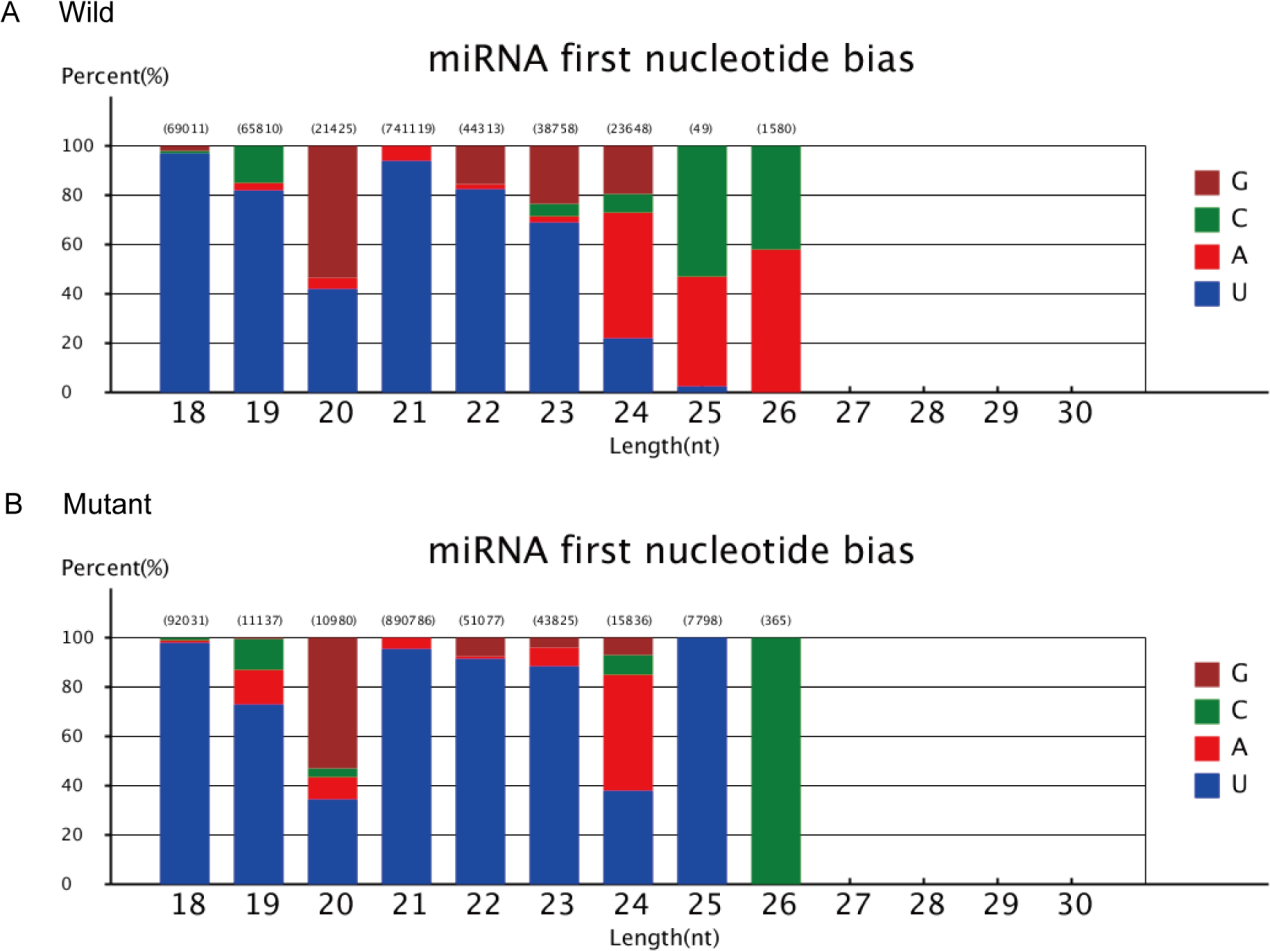
Distribution of miRNA first nucleotide bias. A) wild strain; B) mutant strain.

The identified novel miRNAs could improve wheat miRNA database. The sRNA reads that were homologous to known miRNAs and other non-coding RNAs were excluded. The remaining 20 nt to 22 nt sRNA reads were selected for secondary structure prediction. Novel miRNAs were predicted on the basis of the characteristic hairpin structures of their precursors and sequence specificity (>75% of the reads mapped to the unique locus). A total of 837 and 533 unique miRNA candidates from 497,504 and 483,523 total miRNA candidates in mutant and wild-type strains, respectively, were identified from our small RNA sequencing libraries (Table 5). The different patterns of the miRNA first nucleotide bias were analyzed, which revealed that “U” was the most frequent base at a length of 21, 22, and 23 nt in wild-type and mutant libraries; however, this base was replaced with “G” at a length of 20 nt in both strains (Fig 3 and Table 6). Thus we proposed that some of these predicted novel miRNAs possibly play roles in regulating the plant architecture of wheat. Moreover, our results provided basis to further investigate the regulatory roles of novel miRNAs in wheat production and quality improvements.

**Table 5:** Statistics of predicted novel miRNA precursors.

Samples	Species number of miRNA candidates	Total expression number of miRNA candidates
**mutant**	837	497504
**wild**	533	483523

**Table 6:** Distribution of miRNA nucleotide bias.

	Mutant				wild			
Length	A	U	C	G	A	U	C	G
**18**	1198	89789 (97.56%)	693	351 (0.38%)	117	66760 (96.74%)	740	1394 (2.02%)
**19**	1548	8095 (72.69%)	1406	88 (0.79%)	2137	53641 (81.51%)	9882	150 (0.23%)
**20**	976	3756 (34.21%)	422	5826 (53.06%)	944	8959 (41.82%)	0	11522 (53.78%)
**21**	40510	849414 (95.36%)	834	28 (0.00%)	43764	696413 (93.97%)	919	23 (0.00%)
**22**	723	46512 (91.06%)	0	3842 (7.52%)	871	36417 (82.18%)	0	7025 (15.85%)
**23**	3247	38762 (88.45%)	24	1792 (4.09%)	999	26581 (68.58%)	1911	9267 (23.91%)
**24**	7498	5955 (37.60%)	1200	1183 (7.47%)	12063	5171 (21.87%)	1710	4704 (19.89%)
**25**	22	7763 (99.55%)	2	11 (0.14%)	22	1 (2.04%)	26	0 (0.00%)
**26**	0	0 (0.00%)	365	0 (0.00%)	915	0 (0.00%)	665	0 (0.00%)

### Degradome sequencing and data summary

After RNA was extracted and poly (A^+^) RNA was purified from the wild-type wheat strain Yunong 201 and the mutant strain Yunong 3114, two degradome libraries were constructed and subsequently subjected to degradome sequencing. A total of 20,890,799 (wild) and 18,880,260 (mutant) clean reads were obtained (Table 2) with approximately 99% sequences of 20 nt to 21 nt in length. Two peaks at 20 and 21 nt were observed in length distribution data (Fig 2C and 2D). A total of 5,246,562 (66.61%) unique reads and 15,983,212 (76.51%) total reads in the wild-type strain were matched to the reference genome and 2,881,890 (68.18%) unique reads and 13,865,266 (73.44%) total reads in the mutant strain were matched to the reference genome (Table 3). Using BLASTN search against GenBank and Rfam databases, the structural RNAs (rRNAs, tRNAs, snRNAs, and snoRNAs) were removed (S2 Table), the remaining reads were used to identify conserved and novel miRNAs.

miRNA target genes should be identified and understood to provide insights into the complicated miRNA regulatory network and miRNA-target interactions. Degradome analysis provides large-scale experimental evidence for miRNA-mediated cleavage of target transcripts. A total of 1,207 targets for 434 known miRNAs were obtained in the mutant sample, and 895 targets for 306 known miRNAs were predicted in wild strain (Table 7). To elucidate the functions of novel and known miRNAs in the regulatory network of wheat development, we analyzed the target genes of miRNAs by degradome sequencing. The results showed that 304 targets were predicted to cleave by 96 miRNAs in the mutant strain and 300 targets were predicted to cleave by 73 miRNAs in the wild-type strain (Table 7 and S3 Table). Furthermore, many miRNAs potentially contain multiple targets ranging from one to hundreds whereas some miRNAs had only one target gene. Among the known miRNA families, miR2089 and miR5021 had the highest number of gene targets whereas miR398 had only one target gene. In the newly identified miRNAs, the predicted_miRNA_171 had the largest number of targets.

**Table 7:** Statistics of miRNA targets.

Category	Sample	Numbers of miRNA	Numbers of targets
**Predicted**	Mutant	434	1207
	Wild	306	895
**Degradome evidenced**	Mutant	96	304
	Wild	73	300

We analyzed the annotation of these identified targets and found that >90% of these miRNAs had putative functions; moreover, the targets were enriched in carbon fixation, cytoplasmic part, plastid, cellular metabolic compound salvage, organic substance metabolic process, biosynthetic process, carboxy-lyase activity, and lyase activity (S1 Fig). These results indicated that these novel miRNAs may play an important role in metabolic and biosynthetic processes.

### Specifically and differentially expressed miRNAs

An integrated strategy combining high-throughput sequencing with bioinformatics analyses were used to identify miRNAs. A total of 63 and 35 miRNAs were specifically expressed in wild-type and mutant strains, respectively. Differential expression analysis was also performed to evaluate the miRNA expression profiles on the basis of normalized read count for each known miRNA. A total of 150 miRNAs were identified as significantly and differentially expressed (|fold-change (log_2_ wild/mutant)| > 1, P-value < 0.05); among these miRNAs, 72 were upregulated and 78 were down-regulated in the mutant strain (Table 8). For example, miR867 and miR1077 were upregulated and down-regulated at the highest extent, respectively. However, only seven upregulated miRNAs and 17 down-regulated miRNAs were obtained by degradome sequencing. Moreover, miR5508 and miR5565 showed the most significant fold change. A total of 159 and 432 putative novel miRNAs were specifically expressed in wild-type and mutant strains, respectively (Table 8). Among these novel miRNAs, the predicted_miRNA_733 showed the highest concentration in the wild-type strain; by contrast, the predicted_miRNA_441 was the most abundant in the mutant strain. Simultaneously, 100 putative novel miRNAs were differentially expressed in mutant and wild-type libraries based on the selection criteria of |fold-change (log_2_ wild/mutant)| > 1 between mutant and wild-type strains (S4 Table). Degradome sequencing results showed that four and eight predicted novel miRNAs were upregulated and down-regulated, respectively (Table 8). miRNA expression analysis results revealed that miRNAs could be implicated in wheat growth and development.

**Table 8:** Statistics of specifically and differentially expressed miRNAs.

Expression profiles	Category		Numbers of miRNA by sRNA sequencing	Numbers of miRNA by degradome sequencing evidenced
**Specific**	Mutant	Known	63	13
		New	432	17
	Wild	Known	35	5
		New	159	6
**Up-regulated**		Known	72	7
		New	51	4
**Down-regulated**		Known	78	17
		New	49	8
**Total**				77

**Fig 4:**
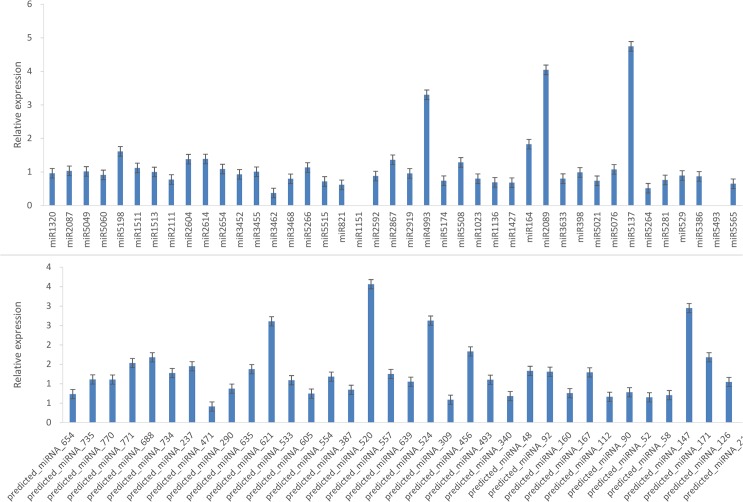
Expression analysis of miRNAs in both wild and mutant strains by qRT-PCR. The expression levels of miRNAs were normalized to the reference gene 5S rRNA.

Moreover, 77 specifically and differentially expressed miRNAs in both strains were selected on the basis of transcriptome sequencing and degradome sequencing results (Table 8 and S5 Table). Real-time PCR was performed to validate the presence of these miRNAs (S1 Fig). Among these miRNAs, miR1151 and miR5493 had very high CG content in their gene sequences to synthesize the primer; thus, these miRNAs were excluded from real-time PCR. The 75 remaining miRNAs, such as predicted_miR_520, miR4993, miR2089, miR5137, miR3462, were expressed by Yunong 3114 and/or Yunong 201 strains (Fig 4). This finding confirmed our sequencing data. Moreover, these 75 miRNAs may be essential for the different phenotypes of Yunong 3114 compared with those of Yunong 201. However, future studies should be conducted to further investigate their functions.

### Identification of miRNA targets

We further analyzed the miRNAs that were significantly and differentially expressed in wild-type and mutant libraries and with targets across small RNA and degradome libraries (S5 Table). Previous research suggested that wheat miRNAs preferably target ubiquitin carrier protein, serine/threonine protein kinase, transcriptional activator Myb, 40S ribosomal protein, F-box/kelch-repeat protein, and BTB/POZ domain-containing protein, which are involved in wheat development [43]. Likewise, we found that the miRNAs predicted in our results target multiple wheat genes, including carboxylase/oxygenase-related proteins (partial chloroplast), photosystem-involved proteins, tubulin-related proteins, ribosomal proteins, transcription factors, and uncharacterized proteins (S5 Table). All of the predicted targets share high homology with *Arabidopsis*, *Oryza*, and *Zea mays*. Most of the predicted targets of the newly identified miRNAs may function in plant growth and development.

## Discussion

### Combined small RNA and degradome sequencing is appropriate for plant miRNA research

As important regulators of gene expression, plant miRNAs change expression during development or in response to environmental stresses. Many researchers applied high-throughput sequencing to identify differentially regulated miRNAs, novel miRNAs, and their targets that modulate development in rice, maize, and wheat [34, 44, 45]. Although studies have been conducted to understand wheat transcriptome, further investigations should be performed to gain insights into their mechanisms and targets. Improvement on crop yield has been the focus in modern agriculture, and crop production is affected by many factors. One of these factors is plant architecture that helps improve grain productivity [15]. Thus, we identified differentially expressed miRNAs in the EMS-treated mutant strain Yunong 3114 and the wild-type strain Yunong 201 by deep sequencing small RNA and degradome libraries to elucidate the epigenetic regulation of the plant architecture of wheat. We also compared the mutant strain Yunong 3114 and the wild-type strain Yunong 201 by the combined high-throughput miRNA profiling and degradome sequencing analysis to identify the miRNAs related to plant architecture. We obtained 10,107,814 (wild) and 11,024,149 (mutant) clean reads by small RNA sequencing. Fragment size distribution was consistent with the results of previous studies [46–48]. For example, a 24 nt class represented 49.99% and 50.93% of the unique reads from wild-type and mutant strains, respectively. The identified precursor miRNA sequences were compared with the previously reported miRNA family; a total of 488 (mutant) and 391 (wild) known miRNAs and 837 (mutant) and 533 (wild) predicted novel miRNA candidates were identified. Small RNA sequencing data showed a good coverage of the known miRNAs. Most of the novel miRNAs identified in the present study started with 5′ uridine, which is a signature of miRNA [49, 50]. A total of 96 (mutant) and 73 (wild) miRNAs were obtained by degradome sequencing. This result indicated that degradome sequencing can be applied to uncover novel functional miRNAs in polyploid plants.

### miRNAs possibly contribute to the development of the relatively preferable plant architecture of Yunong 3114

The number of identified novel miRNAs is higher than that of diploid or tetraploid plants. Diploid plants, such as *Arabidopsis*, rice, maize, and *Populus trichocarpa*, have approximately 325, 592, 172, and 352 miRNAs, respectively. Moreover, tetraploid plants, such as *Medicago truncatula* and *Glycine max*, have approximately 672 and 573 miRNAs, respectively [51]. In the present study, 924 or 1,325 miRNAs were identified in wheat, which is a hexaploid cereal crop. This finding showed that more sets of chromosomes corresponded to more miRNA retention in polyploid. However, rice, which is a high-yield crop, contains a larger number of miRNA than other diploid or even tetraploid plants. This result supported that more miRNAs may produce a high yield of polyploid plants [45]. In the current results, mutation increased the amount of miRNA, which may affect high-yield Yunong 3114; these miRNAs are very potentially related to the development of IPA.

With the development of high-throughput sequencing and advancement in knowledge regarding miRNA functions in plants, studies have revealed that many miRNAs, such as miR156, miR159, miR160, miR162, miR171, and miR172, are conserved and universally expressed among various angiosperms. Previous reports supported the possibility that differentially expressed miRNAs can modulate the level of target mRNA expression and spatial pattern [1]. Therefore, expression profile analysis and predicted target annotation analysis could reveal the significance of miRNAs in plant growth and development. For example, miR156a, miR167d, miR168a, and miR172a are highly expressed in barley grains [52]. Moreover, these miRNAs (except miR172a) are significantly upregulated in developing rice [53, 54] and maize [55] seeds. Furthermore, some reports identified that miR172 is involved in controlling the floral organ identity by regulating the AP2-like genes in rice, maize, and barley [56–58]. miR156 modulates squamosa promoter-binding protein-like substance; moreover, OsSPL14 in rice is regulated by OsmiR156; OsSPL14 contributes to rice plant architecture [17], facilitates panicle branching, and provides higher grain productivity in rice [59]. In addition, miR159, miR165/166, miR396, and miR397 participate in developmental regulation [60–62]. Therefore, these data supported the possibility that miRNAs play significant regulatory roles in plant development and production enhancement of cereal crops.

In our results, very stringent criteria were applied to determine plant architecture-related differentially expressed miRNAs. A total of 150 known miRNAs were identified as significantly differentially expressed between mutant and wild-type libraries. Approximately 72 miRNAs were upregulated and 78 were down-regulated, and these miRNAs may contribute to the plant architecture of the mutant strain Yunong 3114. However, highly abundant miRNAs, such as miR166, miR167, miR168, miR159, miR528, and miR5062, in wild-type and mutant libraries did not show significant fold change. The high-yield mutant strain Yunong 3114 was derived from Yunong 201, which is a high-quality noodle wheat cultivar; thus, the specifically and differentially expressed miRNAs identified in our study may play a major role in Yunong 3114 rather than the known miRNAs, which are related to high yield and plant architecture of other species. A total of 23 miRNAs were specifically expressed in the wild strain whereas 37 miRNAs were specifically expressed in the mutant strain. Among the seven upregulated and 17 down-regulated miRNAs obtained by degradome sequencing, miR5508 (upregulated) and miR5565 (down-regulated) showed the most significant fold change. A total of 159 and 432 putative novel miRNAs were specifically expressed in wild-type and mutant strains, respectively. Approximately 100 putative novel miRNAs were differentially expressed in mutant and wild-type libraries; degradome sequencing data indicated that four (upregulated) and eight (down-regulated) predicted novel miRNAs were possibly related to plant architecture. The amount of miRNAs identified in the mutant library was higher than that in the wild-type library, indicating the important role of mutation in this difference.

In plants, miRNAs target the genes involved in development, particularly transcription factors, metabolic transporters, and signal transduction factors; these genes could unravel a new dimension of miRNA regulatory network [63]. Interestingly, studies have confirmed that miRNA targets are associated with plant development, such as floral organ identity, leaf morphogenesis, root development, and various stress responses in *Arabidopsis* [64, 65]. However, many functions still remain ambiguous. Combined small RNA sequencing, transcriptome sequencing and degradome sequencing promotes the prediction and identification of miRNA targets. Our target prediction results indicated that the putative target of these miRNAs were a set of wheat genes involved in various functions and biological processes, including carbon fixation, cytoplasmic part, plastid, cellular metabolic compound salvage, organic substance metabolic process, biosynthetic process, carboxy-lyase activity, and lyase activity. Differentially expressed miRNAs may regulate target gene expression to affect plant architecture, grain development, and biomass yield in wheat.

Among the predicted targets, several differentially expressed miRNAs (such as miR5508, miR2089 miR5264, predicted_miRNA_48 and predicted_miRNA_171) putatively targeted a set of wheat genes annotated as ribulose-1,5-bisphosphate carboxylase/oxygenase large subunit (rbcL). The accumulation of rbcL products is related to the down-regulation of DNA-binding protease (CND41) in cultured tobacco [66]. Moreover, the reduced CND41 levels induce a high expression level of chloroplast genes, retard senescence, maintain green leaves, and ensure constant protein levels; the reduced CND41 level is also involved in nitrogen translocation [67]. We speculated that the different expression levels of these miRNAs are possibly involved in plant growth and grain development. Few miRNAs, including miR5137, miR5386, miR5493, miR5565, and predicted_miRNA_58, putatively targeted plant photosystem-related genes; thus, these miRNAs are important in the regulation of chloroplast protein synthesis and photosynthesis and in various fundamental biological processes [68]. miR1151 putatively targets F-box-like/WD repeat-containing proteins, which mediate hormone signaling in plants. F-box domain of F-box protein connects protein-protein interaction in various processes, such as polyubiquitination, transcription elongation, centromere binding, and translation repression [69]. Moreover, miR398 putatively targets CSD1, CSD2, and CCS1, indicating that miR398 functions in abiotic and biotic stress response, nutrient homeostasis, and sucrose-specific regulatory pathways [70]. In addition, miR164 targets the NAC family transcription factors, namely, CUC1 (cup-shaped cotyledon1) and CUC2, to regulate petal number [71, 72], age-dependent cell death [73] in *Arabidopsis*, and abiotic and biotic stress response in wheat [37]. miR164 also contributes to the developmental robustness of *Arabidopsis* [74] and may be necessary to establish proper plant architecture in cultivated rice [75]. Furthermore, previous studies suggested that CUC1 and CUC2 regulation by miR164 prevents organ boundary enlargement and formation of extra petals during flower development [76, 77]. Thus, we speculated that the down-regulation of miR164 may also contribute to large grain and strong stalk of the mutant strain Yunong 3114; however, these findings should be further investigated.

Our dataset provides useful information regarding the identified miRNAs; these data may be used to investigate miRNA-mediated regulatory mechanisms in wheat. Our results also suggested that miRNAs contribute to this regulation and determine plant architecture [47]. miRNA targets should be identified and validated as significant steps to reveal the major role of miRNA in the regulatory network of wheat development.

In conclusion, miRNAs and their targets were identified by the combined deep sequencing of small RNA and degradome libraries. A total of 488 and 391 known miRNAs from the wheat mutant strain Yunong 3114 and wild-type strain Yunong 201 were identified, respectively. Moreover, 837 and 533 novel miRNAs were identified from mutant and wild-type libraries, respectively. A total of 304 and 300 genes were identified as cleavage targets of new miRNAs in mutant and wild-type libraries, respectively, by degradome sequencing. Approximately 48 (mutant) and 34 (wild) putative novel miRNAs could be mapped onto the wheat genome. The functions of miRNA targets were also analyzed and discussed. This study provided useful information to further analyze wheat miRNAs and IPA, as well as high yield.

## Supporting Information

S1 Fig GO enrichments of predicted miRNA targets (P-value < 0.05). (TIF)

S1 Table RT-PCR primers of the selected miRNAs. * indicates a high CG content in a particular miRNA sequence; thus, a primer cannot be synthesized. 5S is the reference gene.
(DOCX)

S2 Table Statistics of the unique and total read alignments of wild and mutant strains from GenBank and Rfam databases. (DOCX)

S3 Table miRNA targets confirmed by degradome sequencing. (XLSX)

S4 Table Predicted miRNAs identified as differentially expressed in mutant and wild libraries using the selection criterion of |fold-change (log_2_ wild/mutant)| > 1. (XLSX)

S5 Table Specifically and differentially expressed miRNAs analyzed by the combined RNA sequencing and degradome sequencing. (XLSX)
