# Supplementary figures and images for: Combined Small RNA and Degradome Sequencing Reveals Novel MiRNAs and Their Targets in the High-Yield Mutant Wheat Strain Yunong 3114
Source: PLoS One. 2015 Sep 15;10(9):e0137773. doi: 10.1371/journal.pone.0137773 (PMC4570824; doi:10.1371/journal.pone.0137773)

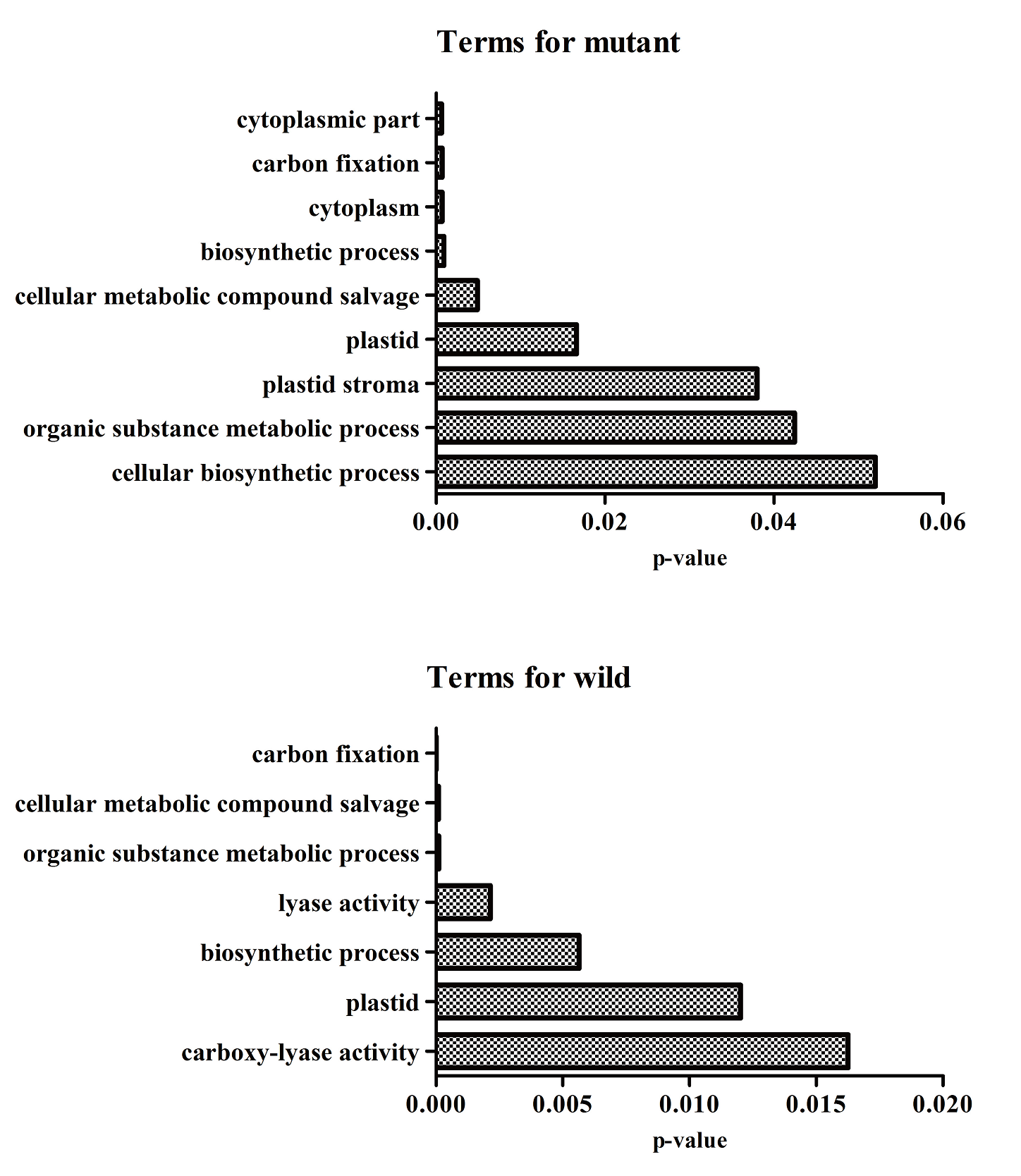

Supplement: S1 Fig — (TIF) [file pone.0137773.s001.tif]
