# Supplementary material for: Combined Small RNA and Degradome Sequencing Reveals Novel MiRNAs and Their Targets in the High-Yield Mutant Wheat Strain Yunong 3114
Source: PLoS One. 2015 Sep 15;10(9):e0137773. doi: 10.1371/journal.pone.0137773 (PMC4570824; doi:10.1371/journal.pone.0137773)
Supplement: S1 Table — * indicates a high CG content in a particular miRNA sequence; thus, a primer cannot be synthesized. 5S is the reference gene. (DOCX) [file pone.0137773.s002.docx]

**Supplementary Data:**

Table S1. RT-PCR primer list of selected miRNAs.

|  | **Name (Universal primer)** | **Primer Sequence** | **Ta(℃)** |
| --- | --- | --- | --- |
| **1** | miR-654 | TAATCTTCTGGATATATGCTTA | 60 |
| **2** | miR-735 | ATTTTGGGTCAGAGGGAGTAC | 60 |
| **3** | miR-770 | ACTTCTTTTGGATCGGGGGGA | 60 |
| **4** | miR-771 | TGGAGGAGGAGAAGGCCTGGT | 60 |
| **5** | miR-688 | TCTTATATTGTGGGGCGGAGT | 60 |
| **6** | miR-734 | TGTGGGAAGGAGGTAGTAGTA | 60 |
| **7** | miR-1320 | TTGGAACGGAGGGAGTATTTTATA | 60 |
| **8** | miR-2087 | GAGAAGGAACCGGCTGCA | 60 |
| **9** | miR-5049 | TCCTAAAATACTTGTCGGGGG | 60 |
| **10** | miR-5060 | GCGGCAAGCTAGAGACAGCAA | 60 |
| **11** | miR-5198 | GGGGAATAGGAGAGGGAG | 60 |
| **12** | miR-237 | TCGAGCTTTGACAGGATGGCG | 60 |
| **13** | miR-471 | CTCGGACCAGGTGTTGAAGAA | 60 |
| **14** | miR-290 | ACTTATTTTGGATCAGAGGGG | 60 |
| **15** | miR-635 | AAACGTTATAGCGCGCCACT | 60 |
| **16** | miR-621 | CAGAGTGGGCATAAGTTCGTC | 60 |
| **17** | miR-533 | GCGCGCTCCTCCGTCGAACG | 60 |
| **18** | miR-605 | TGAGGAATCTGTAGAAGGCGTC | 60 |
| **19** | miR-554 | TCTGCCTGGACGTTTGTAGCA | 60 |
| **20** | miR-387 | TGTTGATCGGGAGGGATGTGG | 60 |
| **21** | miR-520 | TCTTCTATCGTGGGACGAAGG | 60 |
| **22** | miR-557 | ATGCTGGAGCTGGAGCGGCAG | 60 |
| **23** | miR-639 | GGAATGTTGTCTGGTTGGAGA | 60 |
| **24** | miR-524 | CCAGAAGATCGTGGAGTTGCC | 60 |
| **25** | miR-309 | AGGTGGAATACTTGAAGAAGA | 60 |
| **26** | miR-456 | CAAGATGTTGGTGTGCGGTAG | 60 |
| **27** | miR-493 | TGCGTCATGGTTGGGATGGTG | 60 |
| **28** | miR-340 | CAAACGAAGATTGAGGCACCG | 60 |
| **29** | miR-1511 | AACCAGGCTCTGATACCA | 60 |
| **30** | miR-1513 | TATGAGAGAAATCATGACTG | 60 |
| **31** | miR-2111 | GCTCGGAGTCGGCTTATC | 60 |
| **32** | miR-2604 | TAATTTTCGTGTGGGAGTT | 60 |
| **33** | miR-2614 | CGGTTCGGTCTTCGGTTC | 60 |
| **34** | miR-2654 | ATATCAGGGACAAAGGTG | 60 |
| **35** | miR-3452 | TCCCGCGGATTGTCCATG | 60 |
| **36** | miR-3455 | CGGTGGATATGAGGATGTGT | 60 |
| **37** | miR-3462 | GTTCGGTTCTGGGAGCGG | 60 |
| **38** | miR-3468 | TTCGTTTAGGTTGGCGCGT | 60 |
| **39** | miR-5266 | GGGGGACTCTCAGGGGCG | 60 |
| **40** | miR-5515 | TCATGGTTGTTCTAAGGT | 60 |
| **41** | miR-821 | AAATATGAACTAAAAGTGG | 60 |
| **42** | miR-48 | CCACGCTGAGGACTTGCACGG | 60 |
| **43** | miR-92 | TTGTTGTGATGGTCGTCGGAG | 60 |
| **44** | miR-160 | TGCAGGAGTGTTGGGCGGTAC | 60 |
| **45** | miR-167 | TGGGCAGGCAGAAACATTTTT | 60 |
| **46** | miR-2592 | ATGGCTGTTTTGAGGAAGGTA | 60 |
| **47** | miR-2867 | ACAGGACCGTGTGGGATGGCT | 60 |
| **48** | miR-2919 | AAGGGGGGGAGGGGAGAACA | 60 |
| **49** | miR-4993 | GGCGGCTGGTGGAGGATG | 60 |
| **50** | miR-5174 | CTCCGTTCCAAAATAGATGAC | 60 |
| **51** | miR-5508 | TAGATGGCGACGGTAGTGG | 60 |
| **52** | miR-112 | TCATATATTGTGGGGCGGAGG | 60 |
| **53** | miR-90 | TTCGTCGGACGAGCGTGCCT | 60 |
| **54** | miR-52 | TCTTCTATCGTGGGACGGAGG | 60 |
| **55** | miR-58 | AGGAGGGGAGGGAAGGGAAGG | 60 |
| **56** | miR-147 | AGGATGAGTAGGCCACGGGGA | 60 |
| **57** | miR-171 | AGCGCAGAGATGGTCGTCGGT | 60 |
| **58** | miR-126 | TGGATCAGGTACTTGAGGTTG | 60 |
| **59** | miR-214 | ATAGTGAGATAGAGAGAGAGCTG | 60 |
| **60** | miR-1023 | AGGGGATCGTGGAGTGCAT | 60 |
| **61** | miR-1136 | TCGCAGGTATGGATGTATCTA | 60 |
| **62** | miR-1427 | GCGGAACTGTCGGTGGGCGC | 60 |
| **63** | miR-164 | TGGAGAAGCAGGGCACGTGCA | 60 |
| **64** | miR-2089 | AGGATTGGTGTAATGGTA | 60 |
| **65** | miR-3633 | GTGATTGGATGGTTAGGAGG | 60 |
| **66** | miR-398 | TGTGTTCTCAGGTCGCCCCCG | 60 |
| **67** | miR-5021 | AGAGGAAGAAGAAGAAGAAGAA | 60 |
| **68** | miR-5076 | AAATGGGAGCAGAGCAGGTT | 60 |
| **69** | miR-5137 | ACGCGAGAACACGGATGGGCT | 60 |
| **70** | miR-5264 | TTGATCAGGACTTGGCATT | 60 |
| **71** | miR-5281 | TCTTACATTATAGGACGGAGGGAG | 60 |
| **72** | miR-529 | AAAAGAGAGAGAGAAGCAGCC | 60 |
| **73** | miR-5386 | GTCCTCGTCGCCGCGCTG | 60 |
| **74** | miR-5565 | TGTGTGGACTGTTGTCGGC | 60 |
| **75** | miR-5671 | CATGGTGGTGACGGGTGAC | 60 |
| **76** | miR-1151 | CGGGGTGGTCGGACCCGG (*) | 60 |
| **77** | miR-5493 | GCTGGGCTCGGGCGGCGTG (*) | 60 |
| **78** | 5S | GGAGACCGCCTGGGAATA | 60 |
