# Supplementary material for: Combined Small RNA and Degradome Sequencing Reveals Novel MiRNAs and Their Targets in the High-Yield Mutant Wheat Strain Yunong 3114
Source: PLoS One. 2015 Sep 15;10(9):e0137773. doi: 10.1371/journal.pone.0137773 (PMC4570824; doi:10.1371/journal.pone.0137773)
Supplement: S2 Table — (DOCX) [file pone.0137773.s003.docx]

Table S2. Statistics of unique and total reads alignment into Genebank and Rfam databases in wild and mutant strains.

| **Categories** | | **Unique reads** | | | | **Total reads** | | | |
| --- | --- | --- | --- | --- | --- | --- | --- | --- | --- |
|  |  | **Genebank** | | **Rfam** | | **Genebank** | | **Rfam** | |
|  |  | wild | mutant | wild | mutant | wild | mutant | wild | mutant |
| **Small RNA data** | rRNA | 49019 | 33015 | 79042 | 44040 | 702736 | 375987 | 812676 | 339208 |
|  | snRNA | 1204 | 942 | 2555 | 1569 | 6521 | 3709 | 10422 | 4766 |
|  | tRNA | 2612 | 1937 | 14725 | 7880 | 54775 | 27746 | 199359 | 119521 |
|  | other | 3286325 | 4179629 | 3242061 | 4161531 | 9343782 | 10616707 | 9082998 | 10559396 |
|  | total | 3339160 | 4215523 | 3339160 | 4215523 | 10107814 | 11024149 | 10107814 | 11024149 |
| **Degradome data** | rRNA | 13170 | 8930 | 13333 | 7593 | 368160 | 404443 | 128645 | 110984 |
|  | snRNA | 74 | 55 | 2965 | 1416 | 162 | 277 | 5289 | 5394 |
|  | tRNA | 580 | 429 | 2359 | 1409 | 2680 | 3437 | 5095 | 6106 |
|  | other | 7862279 | 4217469 | 7854535 | 4214852 | 20519797 | 18472103 | 20742419 | 18748838 |
|  | total | 7876103 | 4226883 | 7876103 | 4226883 | 20890799 | 18880260 | 20890799 | 18880260 |
